# Psychological, behavioral and social effects of disclosing Alzheimer’s disease biomarkers to research participants: a systematic review

**DOI:** 10.1186/s13195-016-0212-z

**Published:** 2016-11-10

**Authors:** S. A. S. A. Bemelmans, K. Tromp, E. M. Bunnik, R. J. Milne, S. Badger, C. Brayne, M. H. Schermer, E. Richard

**Affiliations:** 1 Department of Neurology, Radboudumc, Geert Grooteplein-Zuid 10, 6525 GA Nijmegen, The Netherlands; 2 Department of Medical Ethics and Philosophy of Medicine, Erasmus MC, Wytemaweg 80, 3015 CN Rotterdam, The Netherlands; 3 Cambridge Institute of Public Health, University of Cambridge, Forvie Site, Robinson Way, Cambridge, CB2 0SR UK

**Keywords:** Alzheimer’s disease, Biomarkers, Disclosure, Risk, Psychological effects, Behavioral effects, Social effects, Prevention studies, Clinical research, Ethics

## Abstract

**Background:**

Current Alzheimer’s disease (AD) research initiatives focus on cognitively healthy individuals with biomarkers that are associated with the development of AD. It is unclear whether biomarker results should be returned to research participants and what the psychological, behavioral and social effects of disclosure are. This systematic review therefore examines the psychological, behavioral and social effects of disclosing genetic and nongenetic AD-related biomarkers to cognitively healthy research participants.

**Methods:**

We performed a systematic literature search in eight scientific databases. Three independent reviewers screened the identified records and selected relevant articles. Results extracted from the included articles were aggregated and presented per effect group.

**Results:**

Fourteen studies met the inclusion criteria and were included in the data synthesis. None of the identified studies examined the effects of disclosing nongenetic biomarkers. All studies but one concerned the disclosure of APOE genotype and were conducted in the USA. Study populations consisted largely of cognitively healthy first-degree relatives of AD patients. In this group, disclosure of an increased risk was not associated with anxiety, depression or changes in perceived risk in relation to family history. Disclosure of an increased risk did lead to an increase in specific test-related distress levels, health-related behavior changes and long-term care insurance uptake and possibly diminished memory functioning.

**Conclusion:**

In cognitively healthy research participants with a first-degree relative with AD, disclosure of APOE ε4-positivity does not lead to elevated anxiety and depression levels, but does increase test-related distress and results in behavior changes concerning insurance and health. We did not find studies reporting the effects of disclosing nongenetic biomarkers and only one study included people without a family history of AD. Empirical studies on the effects of disclosing nongenetic biomarkers and of disclosure to persons without a family history of AD are urgently needed.

**Trial registration:**

PROSPERO international prospective register for systematic reviews CRD42016035388. Registered 19 February 2016.

**Electronic supplementary material:**

The online version of this article (doi:10.1186/s13195-016-0212-z) contains supplementary material, which is available to authorized users.

## Background

Despite numerous studies conducted in the past two decades, there are currently no effective disease-modifying treatments or evidence-based preventive interventions for Alzheimer’s disease (AD) [1]. Because the pathological processes underlying the development of clinical AD are thought to precede the onset of symptoms by years to decades [2–4], previous interventions aiming to prevent AD may have been initiated too late in the disease process. Recently, research initiatives have therefore turned to people who are cognitively healthy but supposedly at increased risk of developing AD on the basis of AD-related biomarkers, in order to slow down or halt the pathological processes and prevent the onset of clinical AD symptoms [5, 6]. Examples of these biomarkers are low amyloid beta 42 and high total or phosphorylated tau levels in cerebrospinal fluid (CSF) [7], positive amyloid PET scans [8] and genetic markers such as the apolipoprotein E (APOE) ε4 genotype [9].

When biomarker examinations are performed in the context of research in people without cognitive complaints, the question arises of whether the results of these examinations should actively be returned to study participants.

Whether active disclosure of biomarker status should be pursued depends on the potential benefit but also potential harm it may cause to research participants. Of all health risks people face in life, many people in both Europe and the USA fear developing AD the most [10, 11]. APOE genetic testing and nongenetic biomarkers such as brain amyloid imaging may indicate an increased risk of developing AD, but do not predict with certainty if and when someone will develop clinical symptoms [12, 13]; the link between, for instance, amyloid positivity and cognitive decline remains particularly elusive [14–16]. Moreover, because there is no disease-modifying treatment or prevention strategy available, the possibility to act upon an increased risk of AD is limited. Based on these considerations, it is plausible that the disclosure of AD-related biomarkers is associated with an unfavorable balance of risks and benefits for some people.

Empirical evidence for the effects of AD genetic susceptibility testing has been reviewed previously [17, 18]. Recent prevention studies do not select individuals solely on the basis of their genotype but also on the basis of nongenetic biomarkers [5, 6]. The impact of disclosing these nongenetic results, which inform research participants of an ongoing pathological process in their brains, is potentially different from the impact of genotype disclosure, which informs of a risk. The effects of disclosing nongenetic AD biomarkers to cognitively healthy research participants have not been reviewed systematically. This systematic review therefore addresses the following question: what are the psychological, behavioral and social effects of disclosing genetic and nongenetic AD-related biomarkers to cognitively healthy research participants?

## Methods

This systematic review is reported in accordance with the Preferred Reporting Items for Systematic Review and Meta-analyses (PRISMA) statement [19].

### Data sources and search strategy

We conducted a systematic literature search in eight electronic databases on April 28, 2015: Embase, Medline, PsycINFO, Cumulative Index to Nursing and Allied Health Literature (CINAHL), Cochrane Central Register of Controlled Trials, Web of Science, PubMed and Google Scholar. The search strategy was developed in collaboration with an information specialist from the Erasmus Medical Centre Medical Library. Search terms used were variations on the key words: dementia, biological markers, genetic testing, disease risk, disclosure, and psychological, behavioral and social factors. The full search strategies for all databases are presented in Additional file 1. Reference lists of included articles were hand searched for additional studies.

### Inclusion and exclusion criteria

**Fig. 1:**
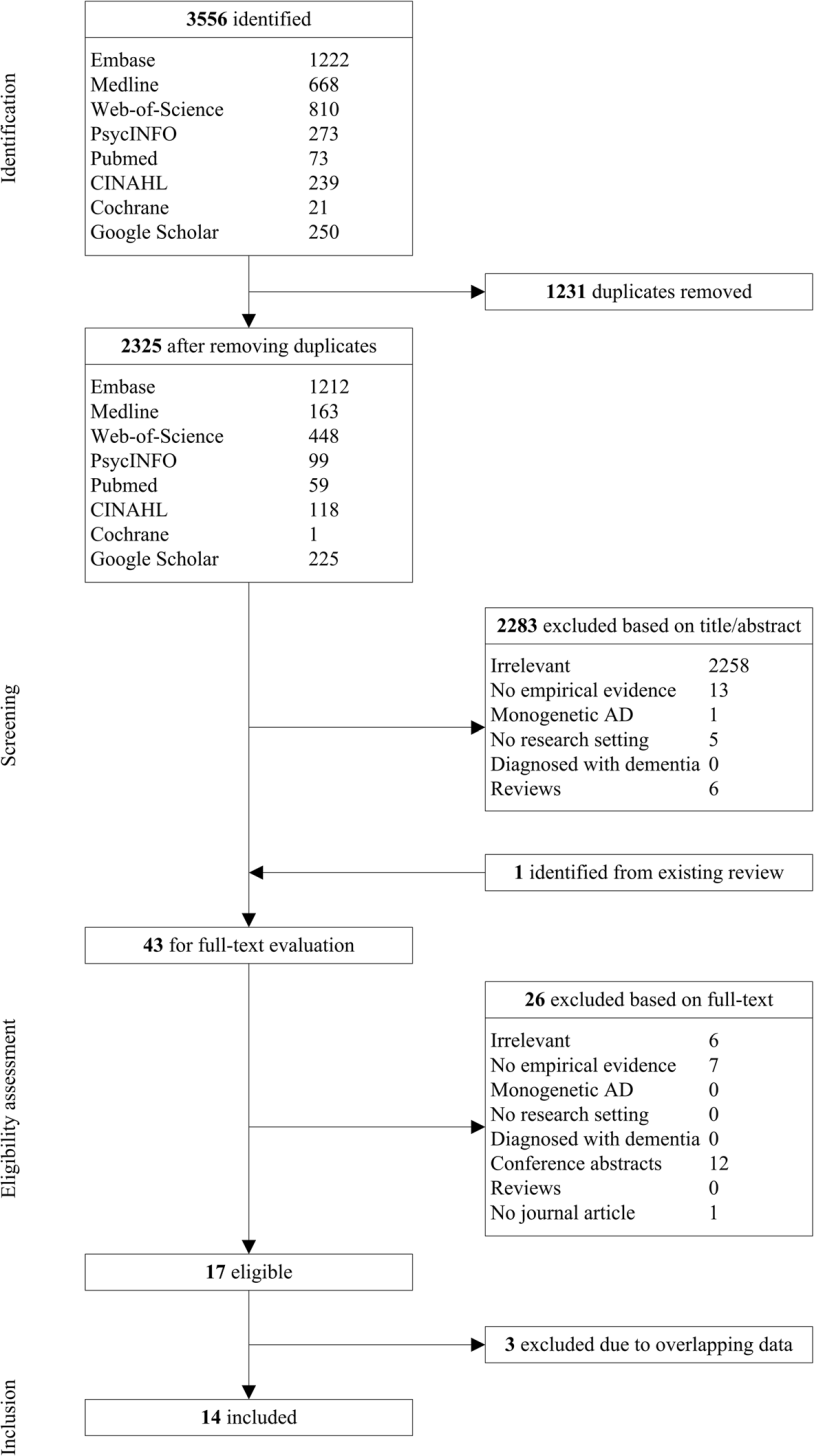
PRISMA flow diagram illustrating process of study selection. In the last step, three studies were excluded due to overlapping data. Fanshawe et al. [53] was excluded because the behavioral outcomes relevant to our research question are also reported in Chao et al. [22]. Cassidy et al. [54] was excluded because the psychological effects relevant to our research question are described more extensively in Green et al. [23] Finally, Roberts et al. [55] was excluded because the psychological effects are described in Green et al. [23] and the behavioral effects in Chao et al. [22] and Zick et al. [24]. *AD* Alzheimer’s disease, *CINAHL* Cumulative Index to Nursing and Allied Health Literature

Articles were included if they: reported on empirical evidence; were published in peer-reviewed journals; were written in English; and reported on actual or hypothetical disclosure. Psychological effects were defined as effects on emotions, mood and cognition. Behavioral effects were defined as changes in behavior that were likely to be caused by the disclosure. Social effects were defined as effects on individuals in their social context. Studies were excluded if they concerned participants who were previously diagnosed with dementia or MCI; or who had a family history of monogenetic AD. If multiple articles reported on the same outcome measures of a single study, the most relevant or extensive report was selected. Excluded studies and reasons for exclusion were documented (see Fig. 1).

### Study selection

Duplicates were removed after retrieving records from the search strategy. In the screening phase, titles and abstracts were independently screened by three reviewers (SASAB, KT and EMB) for eligibility to the research question. In the eligibility phase, full-text articles were assessed by the same three reviewers. In the case of discrepancy between the reviewers, consensus was reached after discussion.

### Data extraction and study quality assessment

Data were extracted from the included studies by one reviewer (SASAB) using a data extraction form (see Additional file 2) that was collaboratively designed and piloted (KT, EMB and ER). We extracted data regarding the main outcome measures (psychological, behavioral and social effects), and additional information on study design, characteristics and population, content and method of disclosure, other outcome measures and funding sources. The risk of bias in quantitative studies was evaluated with the Cochrane Collaboration’s tool for assessing risk of bias, insofar as checklist items were applicable [20]. Qualitative studies were assessed with the Critical Appraisal Skills Programme checklist for quality assessment of qualitative research [21]. Studies with a high risk of bias were excluded from data synthesis.

### Data synthesis

Reported outcomes were both quantitative and qualitative in nature and varied widely between studies, so we composed a metasummary of the reported effects. First, we extracted data on the effects of disclosure from the results sections of the eligible studies. Second, we grouped these effects per outcome measure: psychological, behavioral or social effects.

## Results

### Study selection and quality assessment

A PRISMA flowchart for the results of the study selection is shown in Fig. 1. The electronic database searches generated 2325 unique records. After screening titles and abstracts, 43 records were evaluated in full text for eligibility. After this evaluation, 17 articles were deemed eligible. Three articles were excluded due to overlapping data, because they reported on the same outcome measures of a single study as other included articles. Fourteen articles were eventually identified for data extraction and data synthesis.

For each article, the main potential types and sources of bias were identified (see Additional file 3). All 14 articles assessed were considered not to have a high risk of bias and to be of sufficient quality for inclusion in the data synthesis of this systematic review [22–35].

### Study characteristics of included studies

Of the 14 included studies, two were qualitative studies (interviews) and 12 were quantitative studies. The qualitative studies included 60 and 79 participants [25, 26]. The populations of the quantitative studies varied between 76 and 343 participants, with the exception of one article describing two studies (*n* = 743) [27] and an online survey on hypothetical risk disclosure (*n* = 4036) [28]. In most studies, follow-up assessments were conducted after 6 weeks, 6 months and 12 months. All 14 studies were performed in the USA. Of these 14 studies, 11 reported on data collected in the REVEAL studies. The REVEAL studies are multicenter randomized controlled trials examining the effects of (REVEAL I) and different methods for (REVEAL II and III) APOE genotype disclosure in first-degree relatives (FDRs) of patients with AD. In the REVEAL I study, participants underwent APOE genetic testing and were randomized to receive either their genotype and a lifetime AD risk estimate incorporating this result (the disclosure group) or a risk estimate on the basis of family history, sex and age only (the nondisclosure group) [23]. In the REVEAL II study, everyone received their APOE genotype and participants were randomized to an extended or condensed disclosure protocol with either a face-to-face meeting or an information brochure prior to testing, while both groups had a face-to-face disclosure session [29]. No articles on the actual disclosure of nongenetic biomarkers were identified, although the only article describing hypothetical consequences of disclosure referred to nongenetic biomarker testing [28]. Whereas most studies examined the difference in outcome of interest between those receiving ε4-positive and ε4-negative results, none of them analyzed ε4 heterozygotes and ε4 homozygotes separately. Table 1 presents an overview of the study characteristics.

**Table 1:** Characteristics of studies included in the systematic review

Authors	Year	Country	Study name	Study design	Study population (*n*)	Disclosure	Assessment timepoints	Outcome measures
Studies on psychological effects								
Romero et al. [30]	2005	USA		Cohort	76	APOE genotype	1 month, 4 months, 10 months	Emotional reactions
Gooding et al. [25]	2006	USA	REVEAL QRI	Qualitative	60	APOE genotype	>1 year	Coping
Green et al. [23]	2009	USA	REVEAL I	RCT	162	APOE genotype	Baseline, 6 weeks, 6 months, 1 year	Anxiety, depression, test-related distress
Ashida et al. [31]	2010	USA	REVEAL II	RCT	269	APOE genotype	Baseline, 6 weeks, 1 year	Relation results communication and anxiety, depression and test-related distress
Lineweaver et al. [32]	2014	USA		Case–control	144	APOE genotype	Mean 8.2 months post disclosure	Objective and subjective memory functioning
Green et al. [29]	2014	USA	REVEAL II	RCT	343	APOE genotype	Baseline, 6 weeks, 6 months, 1 year	Anxiety, depression, distress
Studies on behavioral effects								
Zick et al. [24]	2005	USA	REVEAL I	RCT	162	APOE genotype	6 weeks, 6 months, 1 year	Insurance uptake
Chao et al. [22]	2008	USA	REVEAL I	RCT	162	APOE genotype	1 year	Health-related behavior: diet, physical exercise, medication/vitamin intake
Vernarelli et al. [33]	2010	USA	REVEAL II	RCT	272	APOE genotype	6 weeks	Health-related behavior: dietary supplement intake
Taylor et al. [34]	2010	USA	REVEAL II	RCT	276	APOE genotype	Not reported	Insurance uptake
Christensen et al. [27]	2015	USA	REVEAL II/III	RCT	795	APOE genotype	6 weeks, 1 year	Insurance uptake, health-related behavior and other behavior changes
*Hypothetical* Caselli et al. [28]	2014	USA		Survey	4036	Hypothetical disclosure	Not applicable	Anticipated health-related behavior and other behavior changes
Studies on social effects								
Ashida et al. [35]	2009	USA	REVEAL II	RCT	271	APOE genotype	6 weeks	Results communication
Ashida et al. [31]	2010	USA	REVEAL II	RCT	269	APOE genotype	Base, 6 weeks, 1 year	Relation results communication and anxiety, depression and test-related distress
Chilibeck et al. [26]	2011	Canada/USA	REVEAL I	Qualitative	79	APOE genotype	Not reported	Risk perception in relation to family history
*Hypothetical* Caselli et al. [28]	2014	USA		Survey	4036	Hypothetical disclosure	Not applicable	Anticipated results communication

Of the 14 studies included in the systematic review, two report on two types of effects and are taken up twice in this table. Ashida et al. [31] report on both psychological and social effects, and Caselli et al. [28] report on both behavioral and social effects

*APOE* apolipoprotein E, *RCT* randomized controlled trial, *REVEAL* Risk Evaluation and Education for Alzheimer’s Disease Study

### Data synthesis

Of the 14 included studies, six reported on psychological effects, six on behavioral effects and four on social effects (two studies reported on two types of effects). Results from all included studies are summarized in Table 2.

**Table 2:** Results of 14 included studies

Authors, year	Research question/design	Sample (*n*)	Outcome measures	Instruments	Time points	Results			
Studies on psychological effects									
Romero et al., 2005 [30]	Participants were asked how they felt after disclosure of their APOE genotype	76		Self-developed questionnaire		ε4-negative group		ε4-positive group	
			Feeling depressed		1 month	0/49		8/27	
					4 months	0/49		5/27	
					10 months	0/47		4/27	
			Feeling worried		1 month	0/49		6/27	
					4 months	0/49		3/27	
					10 months	0/47		3/27	
			Feeling relieved		1 month	36/49		4/27	
					4 months	30/49		5/27	
					10 months	34/47		3/27	
Gooding et al., 2006 (REVEAL QRI) [25]	56 participants in the REVEAL I study and four individuals who declined participation were interviewed about their reaction to the received results (either risk estimate and genotype or risk estimate only)	60^a^	Coping:	Interviews	>12 months	ε4-positive group		Other (ε4-, ND, not REVEAL-ptc)	
			Relief			4/17 (24 %) were relieved: “Even with the ε4 allele, their risk was not as high as they had previously anticipated”		25/43 felt relieved^b^	
			Worry			Six participants, all ε4+, expressed greater concern about their AD risk after disclosure, describing their results as “depressing”, “frightening” and “disappointing”		14/39 who participated in REVEAL were neither relieved nor worried	
			Neither			7/17 were neither relieved nor worried. They related their “lack of emotion to the lack of predictability of the APOE test and the feeling that it only confirmed what they had already come to accept as their risk for AD”			
Green et al., 2009 [23] (REVEAL I)	Participants were randomly assigned to receive their APOE genotype and a risk estimate (ε4-positive and ε4-negative group) or a risk estimate only (no disclosure group)	162				ε4-positive group	ε4-negative group		No disclosure group
			Anxiety	BAI (0–63)	6 weeks	5.2 (0.7)^c^	4.5 (0.6)		4.4 (0.7)^c^
					6 months	4.6 (0.6)	3.9 (0.6)		4.6 (0.6)
					12 months	4.4 (0.6)	4.2 (0.6)		4.2 (0.6)
			Depression	CES-D (0–60)	6 weeks	9.0 (1.0)	8.5 (0.9)		9.3 (1.0)
					6 months	9.6 (1.0)	8.9 (1.0)		8.7 (1.0)
					12 months	8.3 (0.9)	8.5 (0.9)		8.0 (0.9)
			Distress	IES (0–75)	6 weeks	9.4 (1.3)^d^	5.2 (1.3)		6.7 (1.4)
					6 months	8.6 (1.2)^d^	4.2 (1.2)		8.9 (1.3)^e^
					12 months	8.5 (1.3)	5.1 (1.2)		7.7 (1.5)
Ashida et al., 2010 (REVEAL II) [31]	Participants in the REVEAL II study all received their APOE genotype, and were randomly assigned to either an extended or condensed disclosure protocol	269	Effect of results communication on psychological well-being:	“Have you told anyone your APOE genetic test result? If so, who?”	6 weeks	62.1 % told a family member, 52 % their spouse or significant other, 37.5 % a friend and 14.9 % a health professional			
			Anxiety	BAI	12 months	Telling the result to a friend was associated with a decrease in BAI at 12 months (regression coefficient *b* = –0.17, *p* < 0.01)			
			Depression	CES-D		Telling the result to a healthcare professional with a decrease in CES-D at 12 months (regression coefficient *b* = –0.10, *p* < 0.05)			
			Distress	IGT-AD^f^ (0–60)		There was no association between results communication and IGT-AD levels of distress			
						24 participants (9 %) reported scores > than the clinical cutoff point of 16 on the CES-D at 12 months, and 21 of these 24 had scores < the cutoff at baseline			
Lineweaver et al., 2014 [32]	A group of research participants who were informed about their APOE genotype was compared with a matched group who did not receive their genotype on objective and subjective memory functioning	144	Objective memory functioning Subjective memory functioning	Logical memory subtest	Mean 8.2 months	ε4-positive uninformed	ε4-positive informed	ε4-negative uninformed	ε4-negative informed
				immediate recall		31.9 (8.5)^h^	22.9 (7.8)	28.4 (7.8)	25.6 (5.9)
				delayed recall Wechsler Memory Scale Revised (0–50)^g^		28.1 (9.3)^h^	17.7 (7.9)	24.3 (9.6)	20.6 (6.9)
				Immediate recall delayed recall on Rey–Osterrieth Complex Figure test (0–20)		9.7 (2.7)	10.6 (2.6)	9.3 (3.7)	9.7 (3.1)
						9.0 (3.1)	10.1 (2.4)	9.0 (4.0)	9.9 (2.8)
				Metamemory in Adulthood Questionnaire:					
				Capacity subscale Change over time subscale (15 items 5-point Likert scale)		31.2 (5.2)^i^	28.0 (6.4)	27.8 (6.5)^j^	30.5 (4.7)
						17.0 (5.4)	16.9 (5.5)	15.9 (4.9)	16.7 (4.1)
				Memory Functioning Questionnaire:					
				Retrospective functioning		18.3 (5.8)	15.9 (4.7)	14.3 (4.1)^k^	16.7 (4.7)
				Frequency of forgetting		90.5 (14.7)	83.4 (13.3)	84.5 (17.0)	88.9 (13.4)
				Forgetting when reading		54.9 (8.4)	50.7 (9.3)	50.5 (13.1)^k^	55.5 (8.8)
				Forgetting past events		19.2 (4.6)	18.3 (4.3)	18.1 (4.9)	19.9 (4.5)
				Mnemonics usage (46 items with 7-point Likert scale)		23.1 (11.4)	20.3 (7.4)	22.8 (9.5)	25.1 (8.4)
Green et al., 2014 (REVEAL II) [29]	Participants were randomly assigned to receive their APOE genotype in an extended protocol (SP-GC), a condensed protocol with a genetic counselor (CP-GC) or medical doctor (CP-MD). Aim was to assess whether the condensed protocols were equal (= mean score on any of the scales not more than 5 points higher) to the extended protocol	343				SP-GC	CP-GC		CP-MD
			Anxiety	BAI (0–63)	6 weeks	2.6 (0.5)^c^	3.6 (0.5)		4.3 (0.5)
					6 months	3.2 (0.5)	3.1 (0.5)		4.4 (0.5)
					12 months	3.0 (0.5)	3.7 (0.5)		3.9 (0.5)
			Depression	CES-D (0–60)	6 weeks	5.7 (0.7)	5.8 (0.7)		8.1 (0.7)
					6 months	6.3 (0.7)	5.8 (0.7)		8.1 (0.7)
					12 months	6.2 (0.6)	5.6 (0.6)		6.9 (0.6)
			Distress	IES (0–75)	6 weeks	2.8 (0.9)	5.1 (0.9)		8.2 (0.9)^l^
					6 months	3.9 (0.9)	4.0 (0.9)		7.0 (0.9)^l^
					12 months	3.4 (0.8)	3.3 (0.8)		5.5 (0.8)
Studies on behavioral effects									
Zick et al., 2005 (REVEAL I) [24]	REVEAL I, see Green et al., 2009 [23]	162	Insurance uptake	Questionnaires on actual change in	6 weeks, 6 months, 12 months	ε4-positive group	ε4-negative group		No disclosure group
				Health insurance		12.5	5.56		6.52
				Life insurance		2.08	7.41		6.52
				Disability insurance		4.17	3.70		4.35
				LTC insurance (%)		16.7^m^	1.85		4.35
				Planned change in					
				Health insurance		25.0	13.0		23.9
				Life insurance		16.67	5.56		4.35
				Disability insurance		18.8	7.41		8.70
				LTC insurance (%)		45.8^m^	22.2		32.6
									OR for actual change in LTC insurance ε4+ compared with ND: 5.76 (*p* < 0.1)
Taylor et al., 2010 (REVEAL II) [34]	REVEAL II, see Green et al., 2014 [29]	276	LTC insurance	Not reported	Not reported	Two ε3 traits	≥ one ε4 trait		At least one ε2 trait, no ε4 trait
			OR of actual or planned change			1.00	2.31 (95 % CI 1.11–4.81)		1.55 (95 % CI 0.43–5.60)
			Absolute probability of changing LTC insurance			0.087	0.237		0.149
Chao et al., 2008 (REVEAL I) [22]	REVEAL I, see Green et al., 2009 [23]	162	Health-related behavior changes	Yes/no questions about changes in:	12 months	ε4-positive group	ε4-negative group		no disclosure group
				Any behavior specific to AD prevention		52^n^	24		30
				Medications/vitamins		40	20		28
				Diet		20	11		7
				Exercise (% endorsing)		8	4		5
						OR of any behavior change ε4+ vs ε4– group: 2.73 (95 % CI 1.14–6.54)			
Vernarelli et al., 2010 (REVEAL II) [33]	REVEAL II, see Green et al., 2014 [29]	272	Changes in supplement use	Yes/no questions with free-text field on changes in: overall diet use of dietary supplements exercise (ORs)	6 weeks	ε4-positive group		ε4-negative group	
						1.56 (95 % CI 0.80–3.02)		1.00	
						4.75 (*p* < 0.0001) (95 % CI 2.23–10.10)		1.00	
						1.85 (95 % CI 0.96–3.57)		1.00	
						Of 45 participants reporting a change in supplement use, 32 (71.1 %) were ε4+ (*p* < 0.0001). Of these 45, 38 were in the condensed protocol (84.4 %) and seven in the extended (15.5 %) (*p* = 0.006)			
Christensen et al., 2015 (REVEAL II/III) [27]	Secondary analyses were performed on data from the REVEAL II and III study. For REVEAL II, see Green et al., 2014 [29]. In the REVEAL III study, in-person and phone disclosure, and giving AD genetic info only and pleiotropic info were compared	795	Associations between recruitment status (actively recruited (ARP) or self-referred (SRP)) and behavior changes and advance planning	Yes/no questions on actual and planned changes in:	6 weeks, 12 months	Self-referred participants were more likely than ARPs to report changes to exercise at 12 months (35 % vs 25 %, *p* = 0.032). No other differences between recruitment cohorts were noted on changes or plans to change health behaviors			
				Health behavior: Mental activities Diet Exercise Dietary supplements Medications		Secondary analyses showed that the impact of genetic risk status on certain behavior changes differed by recruitment cohort. ε4-positive participants were more likely than ε4-negative participants to report changes at 6 weeks to mental activities and diet, but only if they had self-referred to the study, although only differences in changes to mental activities persisted through the 12-month follow-up			
				Advance planning: LTC insurance Retirement plans		No direct associations with self-referral were observed on either LTC insurance coverage or retirement plans. An interaction effect was observed (*p* = 0.005): self-referred ε4-positive participants were more likely than ε4-negative participants to report intentions to change LTC coverage, but no differences were noted among ARPs			
						No associations were noted on retirement plans, except greater intentions to change among ε4-positive participants compared with ε4-negative participants, regardless of recruitment cohort (*p* < 0.001)			
*Studies on hypothetical disclosure*									
Caselli et al., 2014 [28]	Members of an online community for people interested in AD prevention research completed a survey on their interest in and anticipated reaction to hypothetical genetic and biomarker testing and disclosure	4036	Results communication	Multiple-choice questions	Not applicable	If APOE ε4 positive, you would tell:		(%)	
						Physician		79.4	
						Spouse		92.3	
						Siblings		84.6	
						Children		81.7	
						Friends		53	
						Lawyer		60	
						If biomarker evidence of AD, you would tell:			
						Spouse		92.2	
						Siblings		80.6	
						Children		75.9	
						Friends		46.5	
						Lawyer		53.8	
			Behavior changes			If APOE ε4 positive, you would:		(%)	
						Begin a healthier lifestyle		90.5	
						Get LTC insurance		76.3	
						Spend all your money forpleasure		18.4	
						Seriously consider suicide		11.6	
						If biomarker evidence of AD, you would:			
						Begin a healthier lifestyle		91	
						Get LTC insurance		76.6	
						Spend all your money for pleasure		18.7	
						Seriously consider suicide		10.2	
Studies on social effects									
Ashida et al., 2009 (REVEAL II) [35]	REVEAL II, see Green et al., 2014 [29]	271	Communication of APOE genetic test result	“Have you told anyone your APOE genetic test result? If so, who?”	6 weeks	Person		Frequency (%)	
						Anyone		81.5	
						Family member		63.8	
						Spouse/significant other		50.9	
						Friends		34.7	
						Health professional		12.2	
						OR of results communication to health professional in condensed vs extended protocol 5.19 (95 % CI 1.50–17.89, *p* < 0.01)			
Chilibeck et al., 2011 (REVEAL I) [26]	Interviews were conducted with participants from REVEAL I and with FDRs of AD patients who did not undergo genetic testing	79 (REVEAL participants) and 40 (non-REVEAL)	Effect of personalized genetic information on conceptualization of personal risk, family health and familial relationships	Open-ended interviews	Not reported	Drawn from the report: when genetic information corresponds with previous beliefs about risk and inheritance, people emphasize how the information provided by genetic testing is “not new” to them but only confirms what they already knew or at least suspected. Yet risk predictions generated by genetic technologies sometimes conflict with those rooted in everyday beliefs about heredity. The visible evidence of risk provided by family history is often more compelling than that based on a genetic test			
						Conclusion: “The genetic risk information given to subjects in the REVEAL trial is interpreted through a process of ‘familiarization’ in which risk estimates are absorbed into and embedded within pre-existing beliefs about who in the family will succumb to AD. These narratives resemble those of individuals from AD families who have not been genetically tested, strongly suggesting that ideas about embodied risk for AD in families are not dramatically changed as a result of genetic testing.”			

Of the 14 studies included in the systematic review, two report on two types of effects. In this table they are taken up only once. Ashida et al. [31] report on both psychological and social effects; results from this study are to be found among the studies on psychological effects in this table. Caselli et al. [28] report on both behavioral and social effects; results from this study are to be found among the studies on behavioral effects in this table

*AD* Alzheimer’s disease, *APOE* apolipoprotein E, *ARP* actively recruited participants, *BAI* Beck Anxiety Inventory, *CES-D* Center for Epidemiological Studies Depression Scale, *CI* confidence interval, *CP-GC* condensed protocol genetic counselor, *CP-MD* condensed protocol medical doctor, *FDR* first-degree relative, *I* informed, *IES* Impact of Event Scale, *IGT-AD* Impact of Genetic Testing for Alzheimer’s Disease, *LTC* long-term care, *ND* no-disclosure group, *OR* odds ratio, *RCT* randomized controlled trial, *REVEAL* Risk Evaluation and Education for Alzheimer’s Disease Study, *SP-GC* standard protocol genetic counselor, *SRP* self-referred participants, *UI* uninformed

^a^Although the question of one’s response to the results is not applicable to those who declined participation in REVEAL (*n* = 4), the original article does not leave those individuals out of this part of the results section

^b^Fifteen participants were in the no-disclosure group in REVEAL and received a lifetime risk estimate only. This group is not analyzed separately in their reaction to the results

^c^Scores are mean values ± SE

^d^Difference ε4+ vs ε4– is significant at 6 weeks and 6 months (*p* < 0.05)

^e^Difference ND vs ε4– is significant at 6 months (*p* < 0.05)

^f^IGT-AD based on the Multidimensional Impact of Cancer Risk Assessment Questionnaire

^g^On all memory scales, higher scores indicate better objective/subjective memory functioning. Scores are mean values ± SD

^h^ *p* ≤ 0.001 ε4+ UI vs ε4+ I

^i^ *p* < 0.05 ε4+ UI vs ε4+ I

^j^ *p* < 0.05 ε4–UI vs ε4– I

^k^ *p* < 0.05 ε4–UI vs ε4– I

^l^Noninferiority of CP-MD vs SP-GC could not be confirmed for test-related distress at 6 weeks and 6 months

^m^ *p* < 0.05 ε4+ vs ε4–

^n^ε4+ vs ε4–, *p* = 0.003; ε4+ vs ND, *p* = 0.03

### Psychological effects

Six studies reported on psychological effects, including anxiety, depression, test-related distress, coping and memory functioning [23, 25, 29–32]. In three studies, anxiety, depression and test-related distress levels were determined with the Beck Anxiety Inventory (BAI), the Center for Epidemiological Studies Depression Scale (CES-D) and the Impact of Event Scale (IES) respectively [23, 29, 31]. One study evaluated coping by means of a self-developed questionnaire [30], and another via interviews [25]. One study assessed objective memory functioning using the Logical memory subtest of the Wechsler Memory Scale Revised (WMS-R) and the Rey-Osterrieth Complex Figure Test (ROCFT), and assessed subjective memory functioning with the Metamemory in Adulthood (MIA) questionnaire and the Memory Functioning Questionnaire (MFQ) [32].

#### Anxiety

REVEAL I showed that, in FDRs of AD patients, there were no differences in anxiety levels between APOE ε4-positive participants, ε4-negative participants and the nondisclosure group [23, 29]. REVEAL II showed that anxiety levels were equal among the different disclosure protocols [29]. In both studies, anxiety levels on the BAI were on average below the clinical cutoff score of 16 [23, 29]. Telling the testing result to a friend was associated with a decrease in anxiety level [31].

#### Depression

REVEAL I showed no differences in postdisclosure depression levels between ε4 carriers, ε4-negative participants and the nondisclosure group [23]. In REVEAL II, depression levels were equal in the different disclosure protocols [29]. In both studies, depression levels on the CES-D were on average below the clinical cutoff point of 16 [23, 29]. Twenty-four REVEAL II participants (9 %) reported depression scores above the clinical cutoff point 12 months post disclosure, and 21 of these 24 had scores below this cutoff score at baseline [29]. Telling the testing result to a healthcare professional was associated with a decrease in depression level [31].

#### Test-related distress

REVEAL I showed that 6 weeks and 6 months, but not 12 months, after disclosure, ε4-positive participants had higher test related distress levels than ε4-negative participants. At 6 months, but not 6 weeks and 12 months, after disclosure, the nondisclosure group had higher test-related distress than ε4-negative participants [23]. In REVEAL II, 6 weeks and 6 months after disclosure, test-related distress levels were higher in the condensed protocol group counseled by a medical doctor compared with the extended protocol group counseled by a genetic counselor. There were no differences in distress between the extended and condensed protocol groups that were both counseled by a genetic counselor [29]. Postdisclosure distress levels were on average below the threshold of 20, indicative of significant distress, in each group [23, 29]. No association was found between results communication and test-related distress levels [31].

#### Coping

One study measured emotional reactions to disclosure 1, 4 and 10 months post disclosure via a self-developed questionnaire: 61–73 % of ε4-negative participants and 11–19 % of ε4-positive participants felt relieved after disclosure. Of the ε4-positive participants, 15–30 % felt depressed and 11–22 % felt worried after disclosure, compared with no individuals in the ε4-negative group [30]. Of the REVEAL participants interviewed in the REVEAL Qualitative Research Initiative, 24 % of ε4-positive participants were relieved after receiving results, versus 58 % of a group consisting of both ε4-negative participants and individuals from the nondisclosure group. Thirty-five percent of ε4-positive participants expressed greater concern about their risk for AD, describing their results as “depressing”, “frightening” and “disappointing”, compared with no one in the other group. Forty-one percent of ε4-positive participants were neither relieved nor worried. In the group consisting of ε4-negative participants and people from the nondisclosure group, 36 % of 39 participants felt neither relieved nor worried after learning their results [25].

#### Memory functioning

One study examined the effects of disclosure on objective and subjective memory functioning. Participants who were aware of being ε4-negative rated multiple aspects of their own memory functioning higher on the MIA and the MFQ than ε4-negative participants who did not know their genotype. Participants who knew they were ε4-positive rated their memory functioning on the capacity subscale of the MIA lower, and performed worse on a logical memory subtest of the WMS-R than ε4-positive participants who were not informed [32].

### Behavioral effects

Six studies assessed one or more behavioral effects, including changes in insurance uptake, health-related behavior and other behavior [22, 24, 27, 28, 33, 34]. Five of those were part of the REVEAL studies [22, 24, 27, 33, 34]. Three studies examined actual and planned changes in insurance uptake within 1 year after disclosure via self-report questionnaires [24, 27, 34]. Health-related behavior changes were assessed by means of yes/no questions on specific behavior changes in three studies [22, 27, 33], and by asking “If APOE ε4-positive/biomarker evidence of AD, you would” followed by several options in a study on hypothetical disclosure [28]. Two studies measured other behavioral effects, one via yes/no questions on specific actual or planned behavior changes [27] and one via the aforementioned question on hypothetical disclosure [28].

#### Insurance uptake

In REVEAL I, in the first year following disclosure, 16.7 % of ε4-positive participants changed long-term care (LTC) insurance, versus 1.9 % of ε4-negative participants and 4.4 % of the nondisclosure group. In addition, 45.8 % of ε4-positive participants planned to change LTC insurance, versus 22.2 % of ε4-negative participants and 32.6 % of the nondisclosure group. No differences were found in changes to health, life or disability insurance uptake [24]. Data from REVEAL II show that ε4-positive participants are 2.3 times more likely to report an actual or planned change to LTC insurance than people with two ε3 alleles [34]. Secondary analyses of REVEAL II data indicate that the intention to change LTC coverage among ε4-positive participants is limited to those who had self-referred to the study [27]. In a study of the hypothetical reaction to disclosure, 76 % would get LTC insurance if APOE ε4-positive and 77 % in the case of (nongenetic) biomarker evidence of AD [28].

#### Health behavior

REVEAL I showed that 12 months after disclosure, ε4-positive participants reported changes in any one of the domains of diet, physical exercise and medication or vitamin intake, more often (52 %) than ε4-negative participants (24 %) or the nondisclosure group (30 %). Within each domain, there were no significant differences between the groups [22]. In REVEAL II, 6 weeks after disclosure, ε4-positive participants were almost five times more likely to report changes to dietary supplement intake, but not to diet or physical exercise, than ε4-negative participants [33]. Secondary analyses of REVEAL II pointed out that ε4-positive participants were more likely to have changed diet 6 weeks after disclosure, mental activities 6 weeks after disclosure and 12 months after disclosure and medication intake 12 months after disclosure than ε4-negative participants, but only if they had self-referred to the study [27]. In a hypothetical disclosure scenario, 90.5 % of respondents would adopt a healthier life style if APOE ε4-positive and 91 % in the case of having (nongenetic) biomarker evidence of AD [28].

#### Other behavioral effects

Two studies reported on other, planned, behavioral effects [27, 28]. The REVEAL II study found greater intentions to change retirement plans among ε4-positive participants than ε4-negative participants [27]. In the study on hypothetical disclosure, if people would be found to be ε4-positive or to have nongenetic biomarker evidence of AD, 18.4 and 18.7 % of respondents would spend all their money for pleasure and 11.6 and 10.2 % agreed to the statement that they would seriously consider suicide [28].

### Social effects

Four studies reported on social effects, namely the communication of results and the perception of risk in relation to family history [26, 28, 31, 35]. Three studies examined results communication; two by asking participants in written questionnaires “Have you told anyone your result? If so, who?” [31, 35]; and one by asking hypothetically “If APOE ε4-positive/biomarker evidence of AD, you would tell …” followed by several options [28]. The results of one of these studies, on the effects communication has on anxiety, depression and test-related distress, are described in the psychological effects section [31]. Risk perception in relation to family history was studied in open-ended interviews [26].

#### Results communication

In REVEAL II, 81.5 % of participants told at least one person their result; 63.8 % told a family member, 50.9 % their spouse or significant other, 34.7 % a friend and 12.2 % a health professional [35]. In a hypothetical scenario of being APOE ε4-positive, 79.4 % of respondents would tell their physician, 92.3 % their spouse, 84.6 % a sibling, 81.7 % their children, 53 % friends and 60 % their lawyer. In case of having (nongenetic) biomarker evidence of AD, 92.2 % would tell their spouse, 80.6 % a sibling, 75.9 % their children, 46.5 % friends and 53.8 % their lawyer [28].

#### Perception

In one study, interviews were conducted with two groups of FDRs of AD patients; the first group consisted of individuals who had undergone genetic testing (REVEAL participants), and the second contained people who had not received information on the genetics of AD [26]. Ideas on the causation of AD and personal risk of developing AD were found to be similar in these groups, suggesting that receiving information on the genetics of AD and undergoing genetic testing for AD does not strongly alter these ideas. The REVEAL participants included in this study considered genetic test results that corresponded with prior beliefs regarding their risk of AD based on family history to be “not new”, whereas if test results conflicted with prior beliefs then family history was often more compelling in participants’ self-perception of risk. Some participants turned out APOE ε4-negative but continued to feel their actual risk was higher than the risk they learned, because of their family history.

## Discussion

In this review on the psychological, behavioral and social effects of disclosing genetic and nongenetic AD biomarkers to cognitively healthy research participants we found that disclosing an APOE ε4 genotype to cognitively healthy FDRs of AD patients in a controlled research context does not appear to result in anxiety or depression [23, 29]. Being informed of ε4-positivity does lead to an increase in test-related distress, LTC insurance uptake and health-related behavior changes when compared with the disclosure of ε4-negativity, and possibly influences memory functioning [22–24, 29, 32–34]. In addition, in people with a family history of AD, disclosure of APOE genotype does not radically alter beliefs regarding the causation of AD and personal risk [26]. No studies reporting on the impact of disclosure of nongenetic biomarkers were identified. Only a single study on hypothetical disclosure referred to both genetic and nongenetic biomarkers.

Based on these studies, disclosure of APOE genotype is often considered to result in relief in case of low risk and to be “safe” in case of high risk [18, 36]. Whereas the first part of this conclusion seems justified [37, 38], several remarks can be made regarding the second.

First, in every study in which genotype was disclosed (except for [32]), participants were FDRs of AD patients. In these individuals the effects of genotype disclosure can be expected to be relatively small, because most may already suspect to be at increased risk on the basis of their family history. Interviews with participants with a FDR with AD show that those with an ε4 allele are indeed not surprised, whereas those without an ε4 allele sometimes find it hard to believe [26]. Hence, results from this particular study population cannot simply be generalized to other groups. In research participants who do not have a family history of AD or who are unaware of the increased risk that is associated with being a FDR of an AD patient, the impact of APOE genotype disclosure may be more substantial. The results of the REVEAL III study, in which a quarter of the participants were not FDRs of AD patients, are expected to show the impact of disclosure in this group and are eagerly awaited [27].

Second, although disclosure does not on average result in more depression, careful interpretation of these results is warranted. In one of the studies, 24 participants (9 %) scored above the clinical cutoff score for depression 12 months after disclosure, while 21 of them scored below this threshold at baseline [31]. In another study, between 15 and 30 % of participants agreed to a statement of feeling depressed after disclosure of an increased AD risk, whereas none of the low-risk individuals did so [30]. It must be noted that these were all participants who underwent psychological screening prior to inclusion, and that it is currently unknown whether the impact of receiving biomarker results is different in people who already experience psychological problems at baseline. These findings suggest that while the impact of disclosure may be low on average, there may be negative consequences for a subset of participants. Although this subset is relatively small, because AD research initiatives aim to recruit large numbers of research participants, a considerable number of people could be negatively affected [5, 6].

Third, disclosure of an increased genetic risk may negatively influence subjective and objective memory functioning in people without cognitive impairment prior to disclosure [32]. Risk disclosure may thus not only lead to a perceived diminishment in memory functioning, but also to a measurable diminishment. A similar effect is known from drug trials in which information on side effects can induce those very symptoms, a phenomenon referred to as the “nocebo” or negative placebo effect. This is supposedly the result of negative expectations regarding the side effects of the intervention [39]. Labeling someone as being at high risk of developing AD may in the same way create negative expectations of one’s own memory functioning, resulting in actual diminished functioning [40].

Fourth, the reported behavior changes after learning one carries an APOE ε4 allele are not merely positive. On the one hand, the increase in LTC insurance uptake reported by ε4-positive participants can be considered a benefit because it corresponds to an important reason why people want to know their AD risk, namely to plan for the future and arrange personal affairs [41–43]. At the same time, fear of insurance discrimination is among the main reasons why people do not want genetic susceptibility testing [41, 42]. Because the increase in LTC insurance uptake by those at increased risk of developing AD indicates a potential for adverse selection [24, 34], which insurers may seek to prevent by charging at-risk individuals more or even refusing them as clients, this fear is not far-fetched; for instance, current US legislation to protect citizens against insurance discrimination on the basis of genetic information does not apply to LTC and disability insurance [44]. Although seemingly innocent, increasing dietary supplement intake may also have negative consequences. It may give people a false sense of control over their situation, as the most frequently taken supplement after genotype disclosure, vitamin E [33], does not help in preventing AD [45]. In fact, it may even have a paradoxically negative effect on cognition in some [46] and has significant potential side effects [47].

The conclusion that disclosing increased genetic risk of AD to healthy research participants is “safe” [18, 36] thus deserves some nuance. The current findings cannot be extrapolated to nongenetic biomarkers because there are potentially important differences [48]. In particular, the presence of nongenetic biomarkers compatible with an “AD profile”, such as a positive amyloid PET scan, is increasingly considered not only as indicative of pathological processes that may eventually lead to the development of AD, but as defining the disease itself, even in the absence of symptoms. In recent research criteria, persons with AD-related nongenetic biomarkers are labeled as being in a “preclinical stage of Alzheimer's disease” [4, 49] suggestive of the inevitable onset of dementia in the future. Although the one study on hypothetical disclosure shows similar envisioned reactions to receiving genetic and nongenetic AD biomarker results, it also shows that one-third of the respondents do not associate (nongenetic) biomarker evidence of AD with either the presence of AD or an increased risk of developing AD [28]. If people receive nongenetic biomarker results in a research context in which they learn that nongenetic biomarkers are not merely risk factors but reflect an ongoing biological process, their response may strongly differ from their reaction to genetic biomarkers.

### Limitations of this systematic review

The present systematic review does not consider risk perception as a psychological effect in itself and because of that the search strategy does not cover this aspect. Gaining a full understanding of the effects of AD biomarker disclosure will need to involve further study of how individual risk perception mediates the information disclosed and psychological or behavioral effects. How one perceives one’s risk may not always be in line with the risk estimate one received and/or recalls [50]. In that case, instead of mediating, the (wrongly) perceived risk may directly cause psychological effects. Finally, risk perception understood in relation to self-perception is an outcome of interest in itself too. Do participants to whom risk is disclosed see themselves the same as before? Or as people who are destined to develop AD? Or maybe even as people who are already ill, waiting for symptoms to manifest [51]?

A second limitation concerns the risk of bias in individual studies. Although we considered all eligible studies of sufficient quality and none as having a high risk of bias, limited bias could have influenced the results. The included studies do not explicitly mention having excluded people with subjective cognitive complaints. An uneven distribution of people with cognitive complaints over the ε4-positive and ε4-negative groups could have interacted with and obscured the relation between the disclosed result and the impact of disclosure. The results of those studies particularly prone to bias (e.g., because of a nonrandomized study design) were and should be interpreted with caution.

Finally, the identified studies had limited heterogeneity, both with regard to the type of biomarker disclosed (APOE genotype only) and with regard to the study population (all US cultural background, mostly FDRs of AD patients and only people without psychological complaints at baseline). Because studies on the public interest in predictive genetic testing for AD show significant cultural differences [52], the effects of disclosure may also differ across cultures and settings. Furthermore, because people with moderate to high levels of anxiety, depression and distress at baseline were excluded from the identified studies, the effect of disclosure on these potentially more vulnerable people, is at present unknown. Therefore, one should be careful in extrapolating the results of these studies to other settings and research participant groups.

## Conclusions

In the context of research, the disclosure of an APOE ε4 genotype overall has no major psychological, behavioral and social impact on cognitively healthy FDRs of patients with AD. The disclosure does, however, lead to elevated test-related distress, depressive symptoms in some, increased uptake of LTC insurance and changes in health-related behavior such as dietary supplement intake. Furthermore, it may affect subjective and objective memory functioning. The impact of disclosing other, nongenetic, biomarkers such as amyloid PET scans or CSF abnormalities is currently unknown. Knowledge of this impact is needed for research projects to decide on whether, when and how biomarker results should be disclosed. Moreover, it will enable institutional review boards to make informed judgments of the risk–benefit ratios in proposed studies, and potential participants in choosing whether or not to participate. Therefore, before engaging in large-scale research projects disclosing biomarkers other than APOE genotype to persons without a family history of AD, research on the psychological, behavioral and social impact of this disclosure is indispensable.

## Additional files

Additional file 1: Includes the full search strategy per electronic database. (DOCX 15 kb)
Additional file 2: A table presenting the data extraction form used for quality assessment of the included studies and data synthesis. (DOCX 14 kb)
Additional file 3: A table presenting the potential for bias in individual studies via an assessment of main potential sources and types of bias per study included in the systematic review. (DOCX 18 kb)

## Abbreviations

AD: Alzheimer’s disease
APOE: Apolipoprotein E
BAI: Beck Anxiety Inventory
CES-D: Center for Epidemiologic Studies Depression Scale
CSF: Cerebrospinal fluid
ε4: Apolipoprotein E ε4 allele
FDR: First-degree relative
IES: Impact of Event Scale
LTC: Long-term care
MFQ: Memory Functioning Questionnaire
MIA: Metamemory in Adulthood
PET: Positron emission tomography
REVEAL: Risk Evaluation and Education for Alzheimer’s Disease Study
ROCFT: Rey-Osterrieth Complex Figure Test
WMS-R: Wechsler Memory Scale Revised
