# Supplementary material for: Psychological, behavioral and social effects of disclosing Alzheimer’s disease biomarkers to research participants: a systematic review
Source: Alzheimers Res Ther. 2016 Nov 10;8:46. doi: 10.1186/s13195-016-0212-z (PMC5103503; doi:10.1186/s13195-016-0212-z)
Supplement: Additional file 1: — Includes the full search strategy per electronic database. (DOCX 15 kb) [file 13195_2016_212_MOESM1_ESM.docx]

**Additional File 1 Full search strategy per electronic database**

**Embase**(dementia/exp OR (dement* OR Alzheimer*):ab,ti) AND (risk/exp OR 'disease predisposition'/de OR 'genetic predisposition'/exp OR 'biological marker'/exp OR 'genetic screening'/exp OR (risk OR risks OR predispos* OR susceptib* OR marker* OR biomarker* OR ((preclinic* OR presymptom* OR predict* OR genetic*) NEAR/3 (test* OR screen*)) OR (pre NEXT/1 (clinic* OR symptom*) NEAR/3 (test* OR screen*)) OR probabilit* OR carrier*):ab,ti) AND ('interpersonal communication'/de OR 'verbal communication'/de OR 'oral communication'/de OR 'written communication'/de OR (disclos* OR communicat* OR ((return* OR reveal* OR give OR giving OR tell* OR notif* OR warn*) NEAR/3 (result* OR informat*))):ab,ti) AND ('psychological aspect'/exp OR 'behavior'/de OR 'behavior change'/de OR 'coping behavior'/exp OR 'defensive behavior'/exp OR emotion/exp OR 'health behavior'/exp OR 'help seeking behavior'/exp OR 'social behavior'/de OR 'emotional attachment'/exp OR 'human relation'/exp OR 'job adaptation'/de OR 'social adaptation'/exp OR 'social attitude'/exp OR 'social bonding'/exp OR 'social cognition'/exp OR 'social interaction'/exp OR 'social participation'/exp OR 'social stigma'/exp OR lifestyle/exp OR 'quality of life'/exp OR 'quality of life assessment'/exp OR stress/exp OR 'psychological wellbeing assessment'/exp OR wellbeing/exp OR Satisfaction/exp OR (psycholog* OR behav* OR consequence* OR impact* OR adapt* OR social* OR emotion* OR relation* OR family-life OR attitude* OR lifestyle* OR (life NEXT/1 style*) OR (quality NEAR/3 life) OR burden* OR harm* OR benefit* OR ((Negativ* OR positiv*) NEAR/3 aspect*) OR stress* OR distress* OR anxi* OR fear* OR anger OR angry OR frustrat* OR mood OR unhapp* OR nervous* OR worry* OR worries OR relief OR relieve* OR solace OR wellbeing OR well-being OR drawback* OR (draw NEXT/1 back*) OR burnout OR agitat* OR implication* OR resilien* OR Satisf*):ab,ti) NOT ([animals]/lim NOT [humans]/lim)

**Medline**(exp dementia/ OR (dement* OR Alzheimer*).ab,ti.) AND (exp risk/ OR exp "Disease Susceptibility"/ OR exp "Biological Markers"/ OR "Genetic Testing"/ OR (risk OR risks OR predispos* OR susceptib* OR marker* OR biomarker* OR ((preclinic* OR presymptom* OR predict* OR genetic*) ADJ3 (test* OR screen*)) OR (pre ADJ (clinic* OR symptom*) ADJ3 (test* OR screen*)) OR probabilit* OR carrier*).ab,ti.) AND (exp "Disclosure"/ OR communication/ OR (disclos* OR communicat* OR ((return* OR reveal* OR give OR giving OR tell* OR notif* OR warn*) ADJ3 (result* OR informat*))).ab,ti.) AND (psychology.xs. OR "behavior"/ OR exp "Behavioral Symptoms"/ OR "Harm Reduction"/ OR "Risk Reduction Behavior"/ OR "Adaptation, Psychological"/ OR exp emotions/ OR exp "health behavior"/ OR exp "social behavior"/ OR life style/ OR exp "quality of life"/ OR "Stress, Psychological"/ OR "Personal Satisfaction"/ OR (psycholog* OR behav* OR consequence* OR impact* OR adapt* OR social* OR emotion* OR relation* OR family-life OR attitude* OR lifestyle* OR (life ADJ style*) OR (quality ADJ3 life) OR burden* OR harm* OR benefit* OR ((Negativ* OR positiv*) ADJ3 aspect*) OR stress* OR distress* OR anxi* OR fear* OR anger OR angry OR frustrat* OR mood OR unhapp* OR nervous* OR worry* OR worries OR relief OR relieve* OR solace OR wellbeing OR well-being OR drawback* OR (draw ADJ back*) OR burnout OR agitat* OR implication* OR resilien* OR Satisf*).ab,ti.) NOT (exp animals/ NOT humans/)

**PsycINFO**(exp dementia/ OR (dement* OR Alzheimer*).ab,ti.) AND (risk factors/ OR exp "Susceptibility (Disorders)" / OR exp "Biological Markers"/ OR "Genetic Testing"/ OR (risk OR risks OR predispos* OR susceptib* OR marker* OR biomarker* OR ((preclinic* OR presymptom* OR predict* OR genetic*) ADJ3 (test* OR screen*)) OR (pre ADJ (clinic* OR symptom*) ADJ3 (test* OR screen*)) OR probabilit* OR carrier*).ab,ti.) AND (communication/ OR (disclos* OR communicat* OR ((return* OR reveal* OR give OR giving OR tell* OR notif* OR warn*) ADJ3 (result* OR informat*))).ab,ti.) AND (exp psychology/ OR exp "behavior"/ OR "Emotional Adjustment"/ OR exp emotions/ OR lifestyle/ OR exp "quality of life"/ OR exp "Stress"/ OR "Satisfaction"/ OR (psycholog* OR behav* OR consequence* OR impact* OR adapt* OR social* OR emotion* OR relation* OR family-life OR attitude* OR lifestyle* OR (life ADJ style*) OR (quality ADJ3 life) OR burden* OR harm* OR benefit* OR ((Negativ* OR positiv*) ADJ3 aspect*) OR stress* OR distress* OR anxi* OR fear* OR anger OR angry OR frustrat* OR mood OR unhapp* OR nervous* OR worry* OR worries OR relief OR relieve* OR solace OR wellbeing OR well-being OR drawback* OR (draw ADJ back*) OR burnout OR agitat* OR implication* OR resilien* OR Satisf*).ab,ti.) NOT (exp animals/ NOT humans/)

**CINAHL**(MH dementia+ OR (dement* OR Alzheimer*)) AND (MH "Disease Susceptibility+" OR MH "Biological Markers+" OR MH "Genetic Screening+" OR (risk OR risks OR predispos* OR susceptib* OR marker* OR biomarker* OR ((preclinic* OR presymptom* OR predict* OR genetic*) N3 (test* OR screen*)) OR (pre n1 (clinic* OR symptom*) N3 (test* OR screen*)) OR probabilit* OR carrier*)) AND (MH "truth Disclosure+" OR MH communication OR (disclos* OR communicat* OR ((return* OR reveal* OR give OR giving OR tell* OR notif* OR warn*) N3 (result* OR informat*)))) AND (MH psychology+ OR MH "behavior+" OR MH "Harm Reduction+" OR MH "Adaptation, Psychological+" OR MH emotions+ OR MH life style+ OR MH "quality of life+" OR MH "Stress, Psychological+" OR MH "Personal Satisfaction+" OR (psycholog* OR behav* OR consequence* OR impact* OR adapt* OR social* OR emotion* OR relation* OR family-life OR attitude* OR lifestyle* OR (life n1 style*) OR (quality N3 life) OR burden* OR harm* OR benefit* OR ((Negativ* OR positiv*) N3 aspect*) OR stress* OR distress* OR anxi* OR fear* OR anger OR angry OR frustrat* OR mood OR unhapp* OR nervous* OR worry* OR worries OR relief OR relieve* OR solace OR wellbeing OR well-being OR drawback* OR (draw n1 back*) OR burnout OR agitat* OR implication* OR resilien* OR Satisf*)) NOT (MH animals+ NOT MH humans+)

**Cochrane**((dement* OR Alzheimer*):ab,ti) AND ((risk OR risks OR predispos* OR susceptib* OR marker* OR biomarker* OR ((preclinic* OR presymptom* OR predict* OR genetic*) NEAR/3 (test* OR screen*)) OR (pre NEXT/1 (clinic* OR symptom*) NEAR/3 (test* OR screen*)) OR probabilit* OR carrier*):ab,ti) AND ((disclos* OR communicat* OR ((return* OR reveal* OR give OR giving OR tell* OR notif* OR warn*) NEAR/3 (result* OR informat*))):ab,ti) AND ((psycholog* OR behav* OR consequence* OR impact* OR adapt* OR social* OR emotion* OR relation* OR family-life OR attitude* OR lifestyle* OR (life NEXT/1 style*) OR (quality NEAR/3 life) OR burden* OR harm* OR benefit* OR ((Negativ* OR positiv*) NEAR/3 aspect*) OR stress* OR distress* OR anxi* OR fear* OR anger OR angry OR frustrat* OR mood OR unhapp* OR nervous* OR worry* OR worries OR relief OR relieve* OR solace OR wellbeing OR well-being OR drawback* OR (draw NEXT/1 back*) OR burnout OR agitat* OR implication* OR resilien* OR Satisf*):ab,ti)

**Web-of-Science**TS=(((dement* OR Alzheimer*)) AND ((risk OR risks OR predispos* OR susceptib* OR marker* OR biomarker* OR ((preclinic* OR presymptom* OR predict* OR genetic*) NEAR/3 (test* OR screen*)) OR (pre NEAR/1 (clinic* OR symptom*) NEAR/3 (test* OR screen*)) OR probabilit* OR carrier*)) AND ((disclos* OR communicat* OR ((return* OR reveal* OR give OR giving OR tell* OR notif* OR warn*) NEAR/3 (result* OR informat*)))) AND ((psycholog* OR behav* OR consequence* OR impact* OR adapt* OR social* OR emotion* OR relation* OR family-life OR attitude* OR lifestyle* OR (life NEAR/1 style*) OR (quality NEAR/3 life) OR burden* OR harm* OR benefit* OR ((Negativ* OR positiv*) NEAR/3 aspect*) OR stress* OR distress* OR anxi* OR fear* OR anger OR angry OR frustrat* OR mood OR unhapp* OR nervous* OR worry* OR worries OR relief OR relieve* OR solace OR wellbeing OR well-being OR drawback* OR (draw NEAR/1 back*) OR burnout OR agitat* OR implication* OR resilien* OR Satisf*)))

**Pubmed publisher**(dementia[mh] OR (dement*[tiab] OR Alzheimer*[tiab])) AND (risk[mh] OR "Disease Susceptibility"[mh] OR "Biological Markers"[mh] OR "Genetic Testing"[mh] OR (risk OR risks OR predispos*[tiab] OR susceptib*[tiab] OR marker*[tiab] OR biomarker*[tiab] OR ((preclinic*[tiab] OR presymptom*[tiab] OR predict*[tiab] OR genetic*[tiab]) AND (test*[tiab] OR screen*[tiab])) OR (pre ADJ (clinic*[tiab] OR symptom*[tiab]) AND (test*[tiab] OR screen*[tiab])) OR probabilit*[tiab] OR carrier*[tiab])) AND ("Disclosure"[mh] OR communication[mh] OR (disclos*[tiab] OR communicat*[tiab] OR ((return*[tiab] OR reveal*[tiab] OR give OR giving OR tell*[tiab] OR notif*[tiab] OR warn*[tiab]) AND (result*[tiab] OR informat*[tiab])))) AND (psychology[sh] OR "behavior"[mh] OR "Behavioral Symptoms"[mh] OR "Harm Reduction"[mh] OR "Risk Reduction Behavior"[mh] OR "Adaptation, Psychological"[mh] OR emotions[mh] OR "health behavior"[mh] OR "social behavior"[mh] OR life style[mh] OR "quality of life"[mh] OR "Stress, Psychological"[mh] OR "Personal Satisfaction"[mh] OR (psycholog*[tiab] OR behav*[tiab] OR consequence*[tiab] OR impact*[tiab] OR adapt*[tiab] OR social*[tiab] OR emotion*[tiab] OR relation*[tiab] OR family-life OR attitude*[tiab] OR lifestyle*[tiab] OR (life ADJ style*[tiab]) OR (quality AND life) OR burden*[tiab] OR harm*[tiab] OR benefit*[tiab] OR ((Negativ*[tiab] OR positiv*[tiab]) AND aspect*[tiab]) OR stress*[tiab] OR distress*[tiab] OR anxi*[tiab] OR fear*[tiab] OR anger OR angry OR frustrat*[tiab] OR mood OR unhapp*[tiab] OR nervous*[tiab] OR worry*[tiab] OR worries OR relief OR relieve*[tiab] OR solace OR wellbeing OR well-being OR drawback*[tiab] OR (draw ADJ back*[tiab]) OR burnout OR agitat*[tiab] OR implication*[tiab] OR resilien*[tiab] OR Satisf*[tiab])) NOT (animals[mh] NOT humans[mh]) AND publisher[sb]

**Google Scholar**dementia|Alzheimer risk|predisposition|susceptibility|probability|carrier disclosure|communication|communicating|" reveal * results" psychological|psychology|behavior|behavioral|consequences|impact|adaptation|emotions|lifestyle|"quality of life"|burden
