# Supplementary material for: Psychological, behavioral and social effects of disclosing Alzheimer’s disease biomarkers to research participants: a systematic review
Source: Alzheimers Res Ther. 2016 Nov 10;8:46. doi: 10.1186/s13195-016-0212-z (PMC5103503; doi:10.1186/s13195-016-0212-z)
Supplement: Additional file 2: — A table presenting the data extraction form used for quality assessment of the included studies and data synthesis. (DOCX 14 kb) [file 13195_2016_212_MOESM2_ESM.docx]

**Additional File 2 Data extraction form**

| Researcher performing data extraction |  |
| --- | --- |
| Date of data extraction |  |
| **Article identification information** |  |
| Author |  |
| Year |  |
| Institute of first author |  |
| Article title |  |
| Journal |  |
| Volume (Issue) |  |
| Pages |  |
| Citation |  |
| Country of origin |  |
| Source of funding |  |
| **Study characteristics** |  |
| Objective |  |
| Design |  |
| Inclusion criteria |  |
| Exclusion criteria |  |
| Recruitment procedure |  |
| Randomization |  |
| Concealment |  |
| **Participant characteristics** |  |
| Number of participants |  |
| Number receiving risk disclosure |  |
| Country |  |
| Age mean (range) |  |
| Gender |  |
| Ethnicity |  |
| Education in years |  |
| Socio-economic status |  |
| Cognitive screening method at baseline |  |
| n and % of potential participants excluded after cognitive screening |  |
| Medical history |  |
| Family history |  |
| Psychological screening method at baseline |  |
| n and % of potential participants excluded after psychological screening |  |
| **Intervention** |  |
| Risk disclosed is based on: |  |
| Information provided on increased risk |  |
| Pre-disclosure education |  |
| Method of disclosure |  |
| What is said about risk to non-disclosure group |  |
| Care received apart from disclosure |  |
| **Outcome measurements** |  |
| Participants at high risk: n (% of total) |  |
| Participants at low risk: n (% of total) |  |
| Outcomes measured |  |
| Measurement scales |  |
| Length of follow-up |  |
| Statistical analysis |  |
| Main results |  |
| Additional measurements |  |
| Number of withdrawals/lost to follow up |  |
| **Critical appraisal** |  |
| Main potential types of bias |  |
| Main potential sources of bias |  |
| **Comments** |  |
