# Supplementary material for: Psychological, behavioral and social effects of disclosing Alzheimer’s disease biomarkers to research participants: a systematic review
Source: Alzheimers Res Ther. 2016 Nov 10;8:46. doi: 10.1186/s13195-016-0212-z (PMC5103503; doi:10.1186/s13195-016-0212-z)
Supplement: Additional file 3: — A table presenting the potential for bias in individual studies via an assessment of main potential sources and types of bias per study included in the systematic review. (DOCX 18 kb) [file 13195_2016_212_MOESM3_ESM.docx]

**Additional File 3 Potential for bias in individual studies**

| **Study and authors** | **Main potential type(s) of bias** | **Potential source(s) of bias and support for judgment** |
| --- | --- | --- |
| **REVEAL I** | | |
| Zick et al. 2005  Chao et al. 2008  Green et al. 2009 | Attrition bias  Performance bias  Reporting bias  Generalisability | - Statistically significant difference between number of subjects withdrawing in nondisclosure group (8, 16%) vs. disclosure group (6, 5%), p=0.04. (Green et al. 2009)  - In communicating APOE Ɛ4-positivity, the susceptibilistic instead of deterministic nature may have been emphasized more than in communicating APOE Ɛ4-negativity, thereby decreasing impact of the former. Risk estimates based on Ɛ4-negativity may have been presented more positively than similar risk estimates without genotype.  - Prespecified secondary outcomes Future Attitudes Scale (FAS) Positive and Negative Affect Schedule (PANAS) were not reported. Health-related behavior and insurance uptake were not prespecified as secondary outcomes. (source: ClinicalTrials.gov nr NCT00571025)  - Subjects limited to individuals with family history of AD, and exclusion of subjects with high baseline anxiety and depression scores |
| **REVEAL II and III** | | |
| Ashida et al. 2009  Ashida et al. 2010  Taylor et al. 2010  Vernarelli et al. 2010  Green et al. 2014  Christensen et al. 2015 | Performance bias  Reporting bias  Attrition bias  Generalisability | - As blinding of personnel to condensed or extended protocol was impossible, participants in the different randomization arms may have (unintentionally) been treated differently in addition to undergoing disclosure in different protocols.  - Primary and secondary outcomes not prespecified for REVEAL II, behavior changes not mentioned as secondary outcomes for REVEAL III. (ClinicalTrials.gov nrs NCT0089882 and NCT00462917)  - In Taylor et al. 2010 and Ashida et al. 2009 it is possible that there was a difference in inclination to change insurance uptake or share results between those withdrawn and those followed-up.  See REVEAL I. |
| **Qualitative studies** | | |
| Gooding et al. 2006 | Selection bias | - "Genetic counselors [...] phoned individuals who they perceived to be willing participants based upon previous interactions during the parent study; [...]" "One hundred and thirty-one individuals were contacted and 56 (43%) agreed to be interviewed." These selection methods may have introduced bias towards interviewing those with a positive disclosure/study experience. |
| Chilibeck et al. 2011 | Selection bias  Other bias | - "Upon the completion of the REVEAL study, 79 participants volunteered to return and take part in open-ended interviews [...]". Possibly those who could not cope well with the information received, or experienced a negative effect on family relations did not wish to participate in these interviews.  - Methods for data collection are described very briefly, data analysis process is not described. |
| **Non-randomized studies** | | |
| Romero et al. 2005 | Reporting bias  Generalisability | - In the methods section the Geriatric Depression Scale (GDS), Profile of Mood States (POMS) and conductance of interviews are mentioned. Outcomes of these assessments and results of interviews are not described in results section.  - Emotional reactions were not measured by comparing emotional states pre- and post-disclosure but by comparing expected emotional response to being at increased risk of AD with actual emotion after either high or low risk disclosure.  - More than half of potential participants were screened out because of psychological reasons such as depression, cognitive impairment, alcohol abuse or life stress. |
| Lineweaver et al. 2014 | Selection bias  Performance bias | - Allocation in part by preference of the participant and by study in which people participated. 15 participants of a study on normal aging who had chosen not to be informed on their genotype were included in the non-disclosure group. The informed participants took part in one of two studies (on normal aging and impact of genetic screening respectively) in which they knew at the time of enrollment that results would be disclosed. It is possible that people who worry about developing AD are more inclined to participate in a study in which they will receive genetic results and respond to disclosure of high risk by performing worse on memory tests more strongly than people who participate in a study in which they may or may not receive genetic testing results.  - As the uninformed and informed participants partially took part in different studies, they have undergone different procedures apart from disclosure/nondisclosure as well. |
| Caselli et al. 2014 |  | - No open questions, e.g. now 18.4% responds positively to the question of spending all their money for pleasure if at increased risk of AD, although it might be the case that very few people would come up with this possibility themselves. |
